# Ocular loiasis affecting a child and its assessment by Anterior Segment Optical Coherence Tomography

**Published:** 2019

**Authors:** Ioan Alexandru Placinta, Camps Isabel Pascual, Cecilia Chiarri-Toumit, Lucía Mata-Moret, Jorge Sanchez-Cañizal, Honorio Barranco-González

**Affiliations:** * University and Polytechnic Hospital la Fe, Valencia, Spain; ** University General Hospital, Valencia, Spain; *** University General Hospital, Valencia, Spain

**Keywords:** Loa loa, subconjunctival, anterior segment optical coherence tomography, AS-OCT, loiasis

## Abstract

A 9-year-old girl from Equatorial Guinea presented to the emergency department complaining of foreign body sensation in her right eye. A thin and large, translucent, slowly moving, coiled worm was observed underneath the conjunctiva. Anterior segment optical coherence tomography revealed hyperreflective small areas surrounded by larger hyporeflective areas into the subconjunctival space. Loa loa microfilaria was evidenced on blood test. Surgical extraction of the subconjunctival worm was intended on slit lamp and under sedation in the operating room, but it was unsuccessful due to poor cooperation and rapid migration of the larva into the sub-Tenon’s space. The patient received two cycles of oral albendazole and one cycle of diethylcarbamazine before achieving complete microfilaria seroconversion.

**Abbreviations:** AS-OCT = Anterior Segment Optical Coherence Tomography, PCR = Polymerase Chain Reaction, DEC = diethylcarbamazine.

## Introduction

Loiasis is a filariasis produced by the *Loa loa* nematode. Tabanid flies belonging to the *Crysops* genus transmit *Loa loa*. Adult parasites live in the subcutaneous tissue causing transient localized inflammatory edema, better known as Calabar swelling and also into the subconjunctival space, while microfilaria larvae are present in the blood stream [**1**,**2**]. It is endemic in West and Central Africa where prevalence in some areas is as high as 50 % [**1**,**2**] and constitutes one of the main reasons for locals to seek medical advice. Two cases of ocular loiasis have been reported so far in Spain [**3**,**4**], three in the USA [**5**-**7**] and other isolated cases in Brazil [**8**], Italy, Norway, Australia, South Korea and the United Kingdom [**9**]. 

More and more cases of ocular loiasis affecting adults are being reported outside of Africa due to an increase in travelling and globalization.

In this report, we present a case of ocular loiasis affecting a child. Anterior segment optical coherence tomography was used to document the subconjunctival filarial worm.

## Case report

A 9-year-old girl presented to the Ophthalmology emergency department with a 3-day history of tingling and foreign body sensation in her right eye.

Her medical and family history was unremarkable, with the exception of her geographic origin: she was born and raised in the Equatorial Guinea, but she had been living in Spain for the past three years. The last visit to Equatorial Guinea was 6 months before. 

**Fig. 1:**
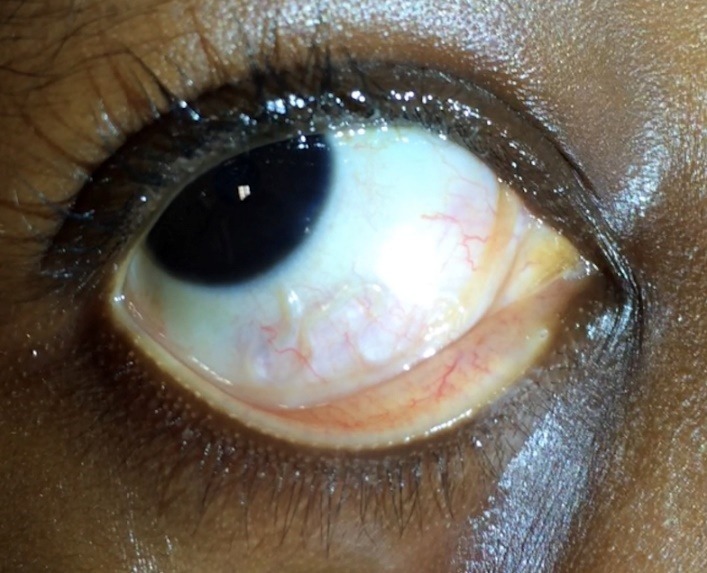
Color photography of patient’s right eye. *Loa loa* larva can be observed at the inferior subconjunctival space

Visual acuity was 20/20 in both eyes. On slit lamp examination, a large, translucent, coiled, slowly moving worm was observed underneath the inferior bulbar conjunctiva on the patient’s right eye (**Fig. 1**). No other pathological findings were observed on anterior and posterior pole examination.

Surgical extraction of the subconjunctival worm was intended on slit lamp, after instillation of 10% phenylephrine, 1% tropicamide, 0,1% tetracaine and 0,4% oxybuprocaine, but it was impossible due to the poor cooperation of our patient. Complete worm paralysis was not achieved. A second attempt was made 20 minutes later, in the operating room, under sedation. By the time of surgery, the larva had already migrated into the retroocular sub-Tenon’s space, making its surgical extraction impossible.

The patient was sent home with a prescription of ciprofloxacin ointment and fluorometholone drops, three times daily each. She was referred to the pediatric ophthalmology clinic the next day.

In clinic, slit lamp examination of anterior and posterior segment revealed no signs of any worm. Ocular motility and pupillary response were conserved with no relative afferent pupillary defect. There were no signs of conjunctival hyperemia, Tyndall or vitritis. A complete blood count and polymerase chain reaction (PCR) for filarial DNA were performed. The patient was advised to come back to the emergency room if any symptoms came back.

**Fig. 2:**
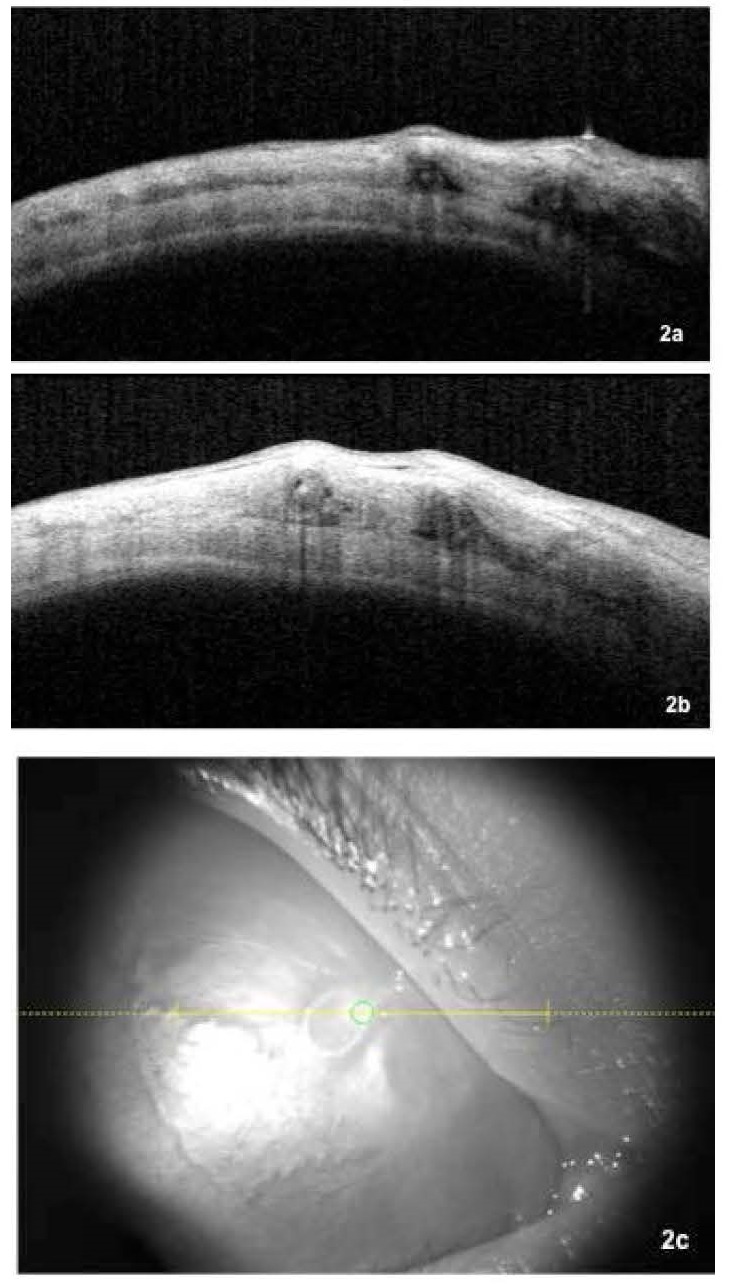
CASIA AS-OCT image composition of right eye. a. Horizontal cross-section (at the level of yellow line in **Fig. 2c**) evidencing hyperreflective areas surrounded by larger hyporeflective areas in the subconjunctival space, corresponding with the *Loa loa* larva; b. Vertical cross-section (perpendicular to yellow cross-section line in **Fig. 2c**) showing hyperreflective areas surrounded by larger hyporeflective areas in the subconjunctival space corresponding with the *Loa loa* larva. c. Super temporal quadrant infrared image showing *Loa loa* larva in the subconjunctival space

**Fig. 3:**
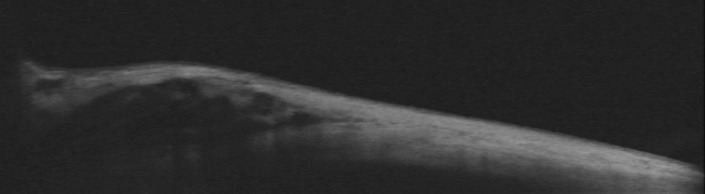
Cirrus AS-OCT image of the right eye

That same evening, she turned back to the emergency room complaining of tingling and the presence of the worm in her right eye. Slit lamp examination confirmed the presence of the worm under the medial bulbar conjunctiva. Again, 10% phenylephrine, 1% tropicamide, 0,1% tetracaine and 0,4% oxybuprocaine were instilled in an attempt of numbing the conjunctiva and paralyzing the worm for eventual surgical extraction. While waiting for proper numbing, anterior segment optical coherence tomography (AS-OCT) was performed to document the larva (**Fig. 2**,**3**).

Vertical cross section at inferior bulbar conjunctiva showing a hyporeflective coiled area in the subconjuntival space, corresponding to the coiled *Loa loa* larva.

On this occasion, the patient’s collaboration was slightly better. Opening of the conjunctiva was achieved under slit lamp visualization, but due to some discomfort, the patient failed to cooperate for further extraction. While preparing for another surgical exploration under sedation, the larva migrated one more time into the deep retroecuatorial sub-Tenon’s space, so the procedure was cancelled.

Next day, the blood test revealed 1180 eosinophils/ µl, 2000 *Loa loa* microfilariae/ ml, and presence of *Loa loa* DNA on PCR sequencing.

Treatment was started with oral albendazole 200 mg twice daily for 3 weeks, obtaining a decrease in blood microfilaremia from 2000 to 450 microfilariae/ ml. One month later, a second cycle of albendazole was administered achieving a reduction of microfilaremia to 150 microfilariae/ ml. Finally, a 3 weeks cycle of oral diethylcarbamazine (DEC), 90 mg three times daily, led to microfilaremia seroconversion. 

New manifestations of the subconjunctival worm were no longer reported since the beginning of the antiparasitic treatment.

The patient did not present Calabar swelling at any time. 

Prednisone (1 mg/ kg/ day) was used prophylactically while on albendazole and DEC, to avoid encephalopathy.

## Discussion

A differential diagnosis had to be established between *Loa loa* [**1**,**2**], *Onchocerca volvulus*, *Mansonela perstans* [**8**] well-known filarial nematodes responsible for subconjunctival filariasis in the Equatorial Africa and *Dirofilaria repens*, an emerging ocular filariasis affecting rural areas of Eastern and Southern Europe, Minor and Central Asia [**10**]. 

Most cases of subconjunctival loiasis described in the literature, affected adults [**3**-**9**]. Surgical extraction of the larva was successful in most of them [**3**-**8**]. Burgués – Ceballos et al. [**3**] achieved a successful extraction under topical anesthesia using 5% lidocaine. Nam et al. [**5**] described the successful immobilization and removal of subconjunctival *Loa loa* larva after injecting the subconjunctival space with 0,5 ml of 2% lidocaine with 1:100,000 epinephrine, whereas Tse et al. [**6**] reported a successful extraction after injecting 1% lidocaine with 1:100,000 epinephrine.

Our patient was a 9-year-old child afraid of sharp instruments around her eye. We were not able to extract the worm using topical anesthesia at the slit lamp, mainly due to the lack of cooperation, which is understandable given our patient’s age. We believe that the primary intent of extraction under sedation, in the operating room, might be a more successful approach in uncooperative children. 

The systemic treatment recommended is DEC 2 mg/ kg three times daily for 3 weeks. More than one cycle may be necessary. A complication of this treatment is the encephalopathy, which can occur in patients with high microfilarial loads (30,000-50,000/ ml). The encephalopathy is produced by endotoxins release and by the cerebral capillaries obstruction with the damaged parasites. In such cases, albendazole is recommended to reduce microfilarial load before using DEC. Ivermectine represents an alternative treatment to DEC but with a higher risk of encephalopathy. Oral prednisone and antihistamine medication may reduce the adverse effects related [**1**,**2**].

## Conclusion

To the best of our knowledge, this is the third report of ocular loiasis in Spain, and the first worldwide, to be assessed by AS-OCT.

*Loa loa* infestation is a rare condition in developed countries; however, due to globalization and migration from endemic areas of Africa, cases are increasingly being reported in this part of the world [**3**-**9**]. Ophthalmologists should be aware of this condition in order to diagnose it correctly. As a new, non-invasive diagnostic tool, AS-OCT may be useful in the assessment of ocular loiasis and related filariasis.

**Compliance with ethical standards**

An informed consent was obtained from the parent of the patient included in the Case Report.

**Acknowledgments**

None.

**Sources of Founding**

None.

**Disclosures**

None. 
